# Perturbation-Theory Machine Learning for Multi-Target Drug Discovery in Modern Anticancer Research

**DOI:** 10.3390/cimb47050301

**Published:** 2025-04-25

**Authors:** Valeria V. Kleandrova, M. Natália D. S. Cordeiro, Alejandro Speck-Planche

**Affiliations:** LAQV@REQUIMTE/Department of Chemistry and Biochemistry, Faculty of Sciences, University of Porto, 4169-007 Porto, Portugal; valeria.kleandrova@gmail.com (V.V.K.); ncordeir@fc.up.pt (M.N.D.S.C.)

**Keywords:** PTML, fragment-based topological design, multi-target drug discovery, de novo drug design, topological indices, anticancer

## Abstract

Cancers constitute a group of biological complex diseases, which are associated with great prevalence and mortality. These medical conditions are very difficult to tackle due to their multi-factorial nature, which includes their ability to evade the immune system and become resistant to current anticancer agents. There is a pressing need to search for novel anticancer agents with multi-target modes of action and/or multi-cell inhibition versatility, which can translate into more efficacious and safer chemotherapeutic treatments. Computational methods are of paramount importance to accelerate multi-target drug discovery in cancer research but most of them have several disadvantages such as the use of limited structural information through homogeneous datasets of chemicals, the prediction of activity against a single target, and/or lack of interpretability. This mini-review discusses the emergence, development, and application of perturbation-theory machine learning (PTML) as a cutting-edge approach capable of overcoming the aforementioned limitations in the context of multi-target small molecule anticancer discovery. Here, we analyze the most promising investigations on PTML modeling spanning over a decade to enable the discovery of versatile anticancer agents. We highlight the potential of the PTML approach for the modeling of multi-target anticancer activity while envisaging future applications of PTML modeling.

## 1. Introduction

Cancers constitute a wide and heterogeneous group of malignant neoplasms that represent a great threat to human life. In 2022, nearly 20 million new cases and 9.7 million deaths were caused by cancers [1]. Two factors of paramount importance prevent the discovery of more efficacious therapeutic solutions against cancers. On one side, many types of cancers are characterized by poor health outcomes, which are directly related to their grade and stage [2]. On the other hand, cancers have an intrinsic multi-factorial nature associated with multiple genetic mutations [3,4,5,6,7]; such biological adaptability provides cancers with the necessary means to evade the immune system, reach metastasized stages, and become resistant to current chemotherapeutic treatments [8]. As a consequence of these two factors, many of the current anticancer medications, which act through one mechanism of action, have become increasingly inefficient in tackling cancer progression [9,10]. All this points to the direction of a paradigm-shifting moment in anticancer research: from single-target modulation to multi-target drug discovery (MTDD) paradigm; the latter has emerged as a more promising option in the sense of providing more versatility, efficacy, and safety [11].

In modern drug development campaigns, in silico approaches have been demonstrated to be essential pillars, accelerating the design of (and/or the search for) novel and versatile molecular entities in multiple MTDD scenarios [12,13]. Particularly, within MTDD-based anticancer research, different in silico approaches have been employed to identify multi-target anticancer agents. These include ligand- and structure-based drug design methods [14,15,16,17,18,19,20,21,22], as well as complex network modeling [15,16,17,20,23]. In addition, predictive models derived from machine learning algorithms have also been applied in the context of either target-focused drug discovery or phenotypic drug search [24,25,26,27,28]. Although all these in silico approaches have been at the forefront of MTDD-based anticancer research, they present one or more of the following major disadvantages. First, some of them have relied on homogenous datasets of chemicals, impeding the search for wider regions of the chemical space. Second, they predict activity by considering only one measure of activity and one biomolecular (e.g., protein) or cellular (i.e., cancer cell line) target, thus neglecting the multi-factorial genetic nature of cancers; consequently, chemicals lacking multi-target activity are unlikely to become effective anticancer drugs. Third, usually, insufficient information is provided on the impact of the diverse assay protocols on the assessment of the different activity measurements; this can create a bias when performing activity prediction and subsequent biological experimentation. Last, in terms of physicochemical and structural interpretability, all the in silico approaches mentioned until now do not offer an accurate rationale on how to design new molecular entities with the desired multi-target anticancer activity.

The in silico approach known as perturbation-theory machine learning (PTML) has emerged to solve all the aforementioned disadvantages [29,30,31,32,33,34]. In this sense, PTML models can simultaneously predict multiple biological effects (activity, toxicity, pharmacokinetics) against dissimilar targets, which include proteins, microbial strains, cell lines, laboratory animals, and humans, among others [29,30,31,32,33,34]; information on different assay conditions have also been considered. Thus, PTML modeling has been applied to the discovery and/or design of molecules against microbial infections [35,36,37,38,39,40,41,42,43,44], neurological disorders [45,46,47,48,49,50], nano-systems for drug release or treatment [51,52,53,54,55,56,57,58,59], and different subfields at the interface of immunology and toxicology [60,61,62,63,64]. In this review, we focus on the emergence, development, and application of PTML modeling for MTDD-based anticancer research in the realm of small-molecule drug discovery. In this sense, we first briefly discuss key concepts and elements of PTML modeling. We then analyze the works that have applied the PTML approach to the modeling of the multi-target anticancer activity of chemicals at both the protein inhibition and phenotypic (cell-based) levels. We also discuss cutting-edge investigations that demonstrate that the physicochemical and structural interpretations of the PTML models can lead to the de novo design of molecules with the desired multi-target anticancer activity. Finally, we envisage some future perspectives on the use of PTML modeling in MTDD-based anticancer research.

## 2. An Overview of PTML Modeling

**Figure 1:**
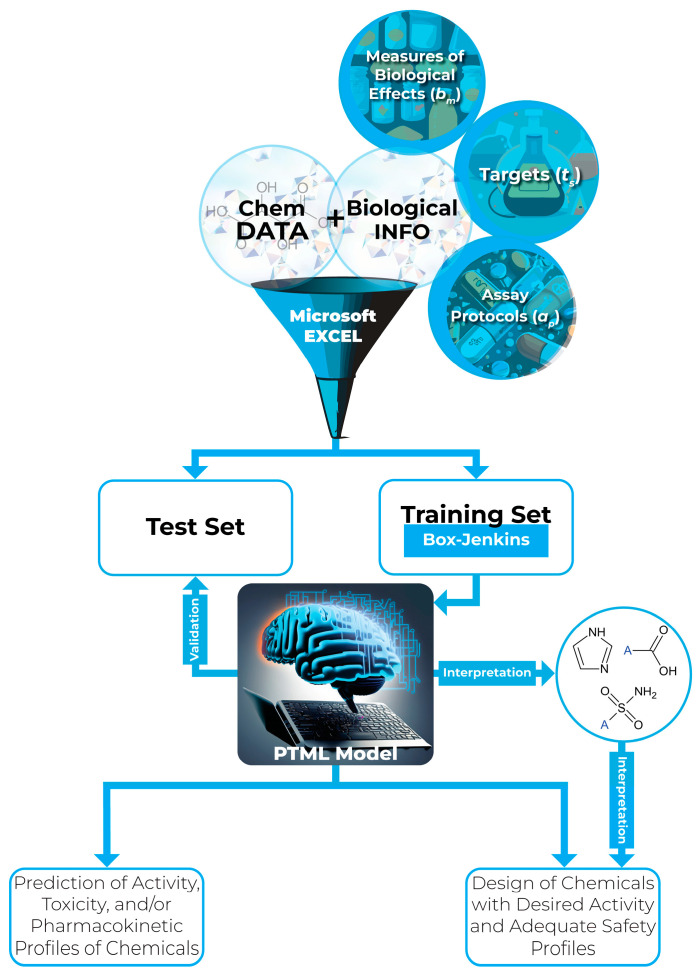
Generation and applications of a PTML model for small-molecule drug discovery. Chemical data related to the molecular structures are usually provided as simplified molecular-input line-entry system (SMILES) codes, which allow the calculation of the molecular descriptors (*X*). Interpretation is carried out by applying the fragment-based topological design (FBTD) methodology, where fragments are analyzed (with A being any atom or functional group).

Currently, PTML models are viewed as advanced models for quantitative structure-activity relationships (QSAR) [29,30,31,32,33,34,35,36,37,38,39,40,41,42,43,44,45,46,47,48,49,50,51,52,53,54,55,56,57,58,59,60,61,62,63,64]. Because the creation and application of PTML models have been discussed in detail elsewhere (Figure 1), we will focus here only on the key aspects.

First, PTML models can simultaneously consider different biological effects (endpoints) at both in vitro and in vivo levels (*b_m_*). Second, while predicting multiple endpoints, diverse targets (*t_s_*) are considered. This means, that, in addition to biomolecules (i.e., cancer-related proteins), all biological systems (e.g., cancer cell lines, subcellular components, and mammals including humans) on which a biological endpoint is determined are regarded as targets.

Third, a wide variety of assay conditions (*a_p_*) can be considered when developing PTML models. Fourth, the essential step before developing any PTML model is the application of the Box-Jenkins approach:

(1) $$avg\left[X\right]e_{j}=\frac{1}{n\left(e_{j}\right)}\times \overset{n\left(e_{j}\right)}{\underset{i=1}{\sum }}X_{i}$$

(2) $$D\left[X\right]e_{j}=\left(\frac{X-avg\left[X\right]e_{j}}{Num}\right)\times p{\left(e_{j}\right)}^{Y}\;$$

Notice that in Equations (1) and (2), *X* is a molecular descriptor; this can be an experimentally determined property or a theoretical value calculated from the 2D or the 3D structure of a molecule [65]. Also, *e_j_* refers to an experimental condition, which is a combination of *b_m_*, *t_s_*, and *a_p_*. On the other hand, in Equation (1), we have the average value *avg*[*X*]*e_j_* and *n*(*e_j_*); the latter indicates the number of chemicals that comply with a specific experimental aspect of *e_j_*. Thus, for instance, if *e_j_* = *b_m_*, then, *n*(*e_j_*) = *n*(*b_m_*), with *n*(*b_m_*) indicating the number of molecules experimentally tested by measuring the same biological effect/endpoint *b_m_*. The same reasoning is applied to the elements *t_s_* and *a_p_*. This means that Equation (1) is applied to *b_m_*, *t_s_*, and *a_p_*, separately. It is important to highlight that because many chemicals/molecules are tested in diverse experimental conditions *e_j_*, the values *avg*[*X*]*e_j_* and *n*(*e_j_*) will be different across the different biological endpoint measures (*b_m_*), targets/biological systems (*t_s_*), and assay conditions/protocols (*a_p_*). In Equation (2), the term “*Num*” can be the range (difference between the maximum and minimum values of *X*), the standard deviation of *X* values, or a value of 1. At the same time, *p*(*e_j_*) is an *a priori* probability of finding a chemical tested by considering a specific experimental aspect of *e_j_* (*b_m_*, *t_s_*, or *a_p_*); the exponent *Y* usually is equal to −1, −0.5, 0, 0.5, or 1. It should be highlighted that in Equations (1) and (2), *avg*[*X*]*e_j_*, *n*(*e_j_*), and *p*(*e_j_*) are calculated exclusively from the chemicals/molecules annotated to belong to the training set. The most important term of Equation (2) is *D*[*X*]*e_j_*, which is known as a multi-label index (*MLI*). In this sense, the multi-label indices *D*[*X*]*e_j_* (*MLIs*) are used as inputs for the creation of the PTML models; they maintain the same physicochemical and structural meaning as *X* while also fusing the chemical information (contained within *X*) with the different biological aspects of *e_j_*. The *MLIs* are the ones that allow PTML models to simultaneously predict activity, toxicity, and/or pharmacokinetic profiles of molecules under dissimilar experimental conditions *e_j_*.

A fifth important aspect of PTML modeling is that the use of Equations (1) and (2) allows the classification of PTML models into different categories. If Equations (1) and (2) are employed only in the case of the experimental aspect *t_s_*, then, we will be in the presence of a multi-target QSAR (mt-QSAR) model. If all the experimental aspects of *e_j_* (*b_m_*, *t_s_*, and *a_p_*) are used to simultaneously predict activity, toxicity, and pharmacokinetics, the PTML model will be classified as a multi-tasking model for quantitative structure-biological effect relationships (mtk-QSBER). Any PTML model will be classified as multi-condition QSAR (mtc-QSAR) when using *b_m_*, *t_s_*, and *a_p_* to predict only activity endpoints; the term PTML can be used either instead of mtc-QSAR or any other case. Sixth, as commented in the upcoming section, PTML models are based on machine learning algorithms, particularly artificial neural networks (ANN) and linear discriminant analysis (LDA); however, random forests (RF), support vector machines (SVM), and other algorithms can also be easily implemented to generate PTML models. Seventh, the PTML models are created from the training set and validated by using the test set. Last, because of their ability to predict a multitude of endpoints, PTML models can be very useful as both virtual screening tools and guides for de novo molecular design.

## 3. PTML Models for MTDD-Based Anticancer Research

### 3.1. Key Aspects of the Analysis of PTML Modeling for MTDD-Based Anticancer Research

In the upcoming subsections, we have reported cutting-edge investigations devoted to the development of PTML models for MTDD-based anticancer research. In this sense, we would like to point out the following key aspects. In the discussion, when referring to a specific PTML modeling, we have used a notation that includes the type of PTML model and the machine learning algorithm used to create it. For instance, if a PTML model classified as mt-QSAR was based on the LDA algorithm, then, the notation for that model was “mt-QSAR-LDA”. On the other hand, we have mentioned the type of molecular descriptors (e.g., topological indices, fragment-based descriptors, physicochemical properties, etc.) that were used to calculate the *MLIs* (see Equations (1) and (2)); the main software used to calculate such molecular descriptors were MODESLAB v1.5 [66], DRAGON v5.3 [67] or above, and QUBILs-MAS v1.0 [68,69,70]. During the analysis of the different PTML models, we have mentioned the numbers of the biological effect/endpoint measures and targets employed in the experiments; when considered relevant, assay protocols and other experimental aspects have also been mentioned. We have also reported the number of statistical cases or data points (e.g., molecule/target, molecule/endpoint measure/target, or molecule/endpoint measure/target/assay protocol combinations) in each dataset used to generate the PTML models. It should be highlighted, that, before creating the PTML models, when splitting the datasets into training and test sets, 3:1 or 4:1 splitting ratios were used. This means that the training sets used to create the PTML models were formed by up to 80% of their corresponding datasets; test sets contained at least 20% of the data and were used to validate the PTML models. We have also reported the statistical performance of the PTML models. In doing so, because the statistical performance metrics (SPM) varied across different works, we applied the normalized terminology SPM > v_sm_. Notice that SPM was *Sn* (sensitivity), *Sp* (specificity), or *Acc* (accuracy) while v_sm_ was the value of that particular SPM; the terminology SPM > v_sm_ (or a written similar phrase) meant that for a specific SPM, a value higher than v_sm_ was reported for both training and test sets. The search for PTML models mentioned in the upcoming subsections was performed by the STATISTICA software up to version 13.5.0.17 [71].

### 3.2. The PTML Approach for Modeling of Multi-Target Anticancer Activity

**Figure 2:**
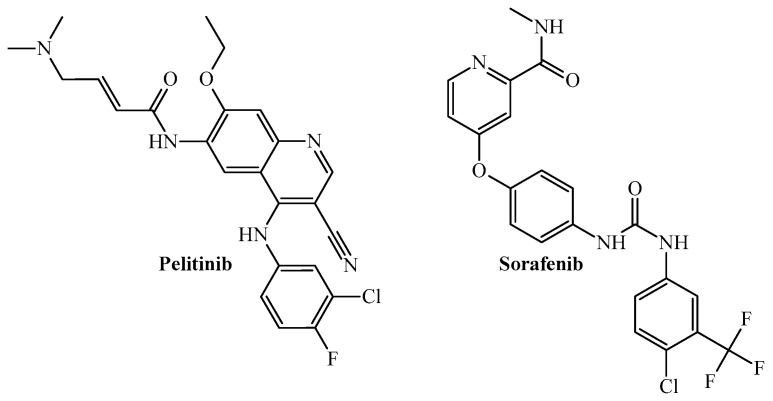
Chemical structures as the two most powerful versatile kinase inhibitors identified by the mt-QSAR-LDA model.

The first report using PTML modeling to predict multi-target anticancer activity dates back to 2011. In this work, the *MLIs* used to build several mt-QSAR-LDA models were derived from the topological indices known as bond spectral moments; the purpose of each model was to simultaneously predict inhibitory activity against eight tyrosine kinase proteins associated with the emergence and/or progression of different types of cancer [72]. The mt-QSAR-LDA models were developed from a dataset containing 1771 cases; five activity cutoff values based on the half-maximal inhibitory concentration (IC_50_) were used, namely 0.05 µM, 0.1 µM, 0.5 µM, 1 µM, and 5 µM. All the mt-QSAR-LDA models exhibited *Sn* and *Sp* values higher than 75%. The best mt-QSAR model was the one based on the IC_50_ cutoff of 0.1 µM. This model was used to predict two small datasets, one formed by 13 molecules reported in the scientific literature and assayed against seven of the eight tyrosine kinases and the other containing three previously synthesized but newly tested quinazoline derivatives. The best mt-QSAR-LDA model satisfactorily predicted both datasets, demonstrating its capacity to accelerate the identification of multi-target tyrosine kinase inhibitors against cancers. Particularly, multi-target anticancer agents such as pelitinib and sorafenib (Figure 2) were predicted by the mt-QSAR-LDA model to exhibit versatile anti-kinase inhibitory activity, thus confirming previous experimental findings.

Another work reported the development of an mt-QSAR-LDA model that was devoted to predicting the multi-cell inhibitory potency of chemicals against 12 sarcoma cell lines [73]. This mt-QSAR-LDA model was generated from 3017 cases using *MLIs* based on bond spectral moments combined with fragment descriptors as inputs. The mt-QSAR-LDA model achieved *Sn*, *Sp*, and *Acc* values above 90%. The mt-QSAR-LDA model enabled the calculation of quantitative contributions of different molecular fragments to the multi-cell anticancer activity against the 12 sarcoma cell lines. It is important to highlight that this work was the first report providing a rationale for the relation of the multi-target phenotypic (cell-based) activity and the chemical structure at the fragment/functional group level.

In a 2014 study, an mtk-QSBER-LDA model was obtained from *MLIs* based on the bond spectral moments to simultaneously predict multi-target activity against proteins associated with bladder cancer and multi-cell anticancer activity against several bladder cancer cell lines, as well as multiple in vitro and in vivo toxicity and pharmacokinetic endpoints against dissimilar targets [74]. The mtk-QSBER-LDA model was constructed from a dataset formed by 39,198 cases, which included 16 diverse endpoints *b_m_*, 49 dissimilar targets *t_s_*, and three different levels of curations/reliability of the assay protocols. The mtk-QSBER-LDA model displayed *Sn* and *Sp* above 95%. Through the calculation of the quantitative contributions, several molecular fragments were identified to simultaneously influence the increment of the multi-protein and multi-cell anticancer activities as well as the reduction of the toxicity and the enhancement of the pharmacokinetic profiles. This work, in addition to generalizing the calculation of quantitative fragment contributions beyond the activity domain (quantitative contribution of fragments were also performed for toxicity and pharmacokinetic profiles), is the only report that has successfully attempted to predict multi-protein/multi-cell anticancer activity while also considering toxic effects and pharmacokinetics.

Nowadays, it is recognized that the ubiquitin-proteasome pathway plays an important role in cancer [75,76,77]. Therefore, in 2015, research was conducted to predict multi-target inhibitors of the ubiquitin-proteasome pathway [78]. Here, an mtc-QSAR-LDA model was built from *MLIs* derived from linear indices and a dataset comprising 5602 cases distributed across 20 different *b_m_*, at least 20 *t_s_*, and 474 assay protocols *a_p_*. The mtc-QSAR-LDA model exhibited *Sn* and *Sp* values higher than 70%. This work constitutes the first attempt to model the multi-target activity of chemicals in the context of MTDD-based anticancer research associated with the ubiquitin-proteasome pathway.

A 2018 study reported the development of two PTML-LDA models for the prediction of multi-protein and multi-cell activities of chemicals against diverse cancers [79], including (but not limited to) those of the breast, ovary, colon, lung, and prostate, as well as melanoma. In this research, the global physicochemical properties known as the logarithm of the n-octanol/water partition coefficient (logP) and polar surface area (PSA) were used to generate the *MLIs*, which subsequently served as inputs for the PTML-LDA models. By employing a dataset containing more than 100,000 cases (involving > 70 *b_m_*, > 300 biomolecular and non-biomolecular *t_s_*, and 4 labels of general assay protocols *a_p_*, among other experimental considerations). Both PTML-LDA models achieved *Sn* > 70% and *Sp* > 89%. The best of the two PTML models was used to carry out simulations on 115,000 data points, enabling the detection of tendencies on multi-target and multi-cell anticancer activity across many assay conditions and their corresponding associations with chemical structure variability.

Last, an investigation was carried out by creating several PTML-LDA and PTML-ANN models for the prediction of the multi-protein and multi-cell activity of chemicals against sarcoma [80]. Here, for the creation of these PTML models, *MLIs* derived from the global physicochemical properties logP and PSA were used as inputs. The dataset used by these PTML models consisted of >37,900 cases distributed across 155 endpoints *b_m_* and 79 biomolecular and cellular targets *t_s_*; in addition, 17 assay organisms were reported as relevant labels during the creation of the PTML models. In this work, the PTML models achieved *Sn* > 79% and *Sp* > 95%. When compared to classical machine learning models employing larger numbers of molecular descriptors, it was concluded that the developed PTML models were considerably simpler yet more informative than their classical counterparts. This work also offered a brief guideline on how to use the different PTML models to perform virtual screening of chemicals with potential multi-protein and/or multi-cell anti-sarcoma activity.

### 3.3. PTML Modeling for De Novo Drug Design in MTDD-Based Anticancer Research

The applications of the PTML approach are not limited only to the modeling of the multi-target anticancer activity; PTML modeling can also be applied to de novo drug design [81,82,83,84]. We would like to highlight that de novo drug design is devoted to generating novel molecules from scratch by using atoms/fragments as building blocks [81,82,83,84]; this means that no starting templates (e.g., known molecular scaffolds) are used.

**Figure 3:**
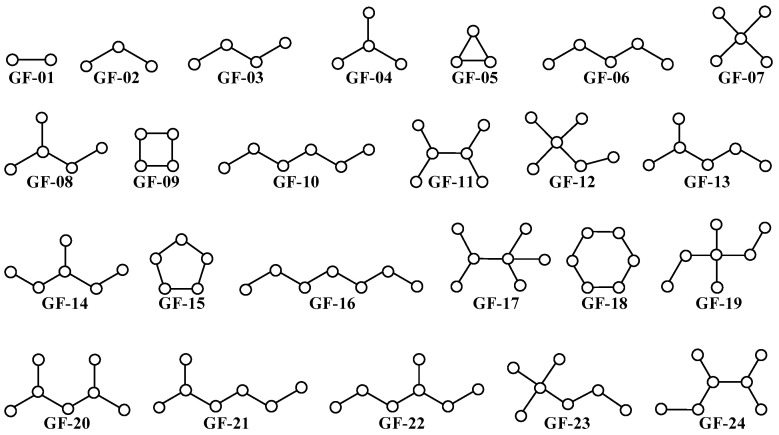
A non-exhaustive list of subgraphs/generic fragments (GF) used by the topological indices to characterize the chemical structure of the molecules. For instance, GF-05, GF-09, GF-15, and GF-18 characterize the presence of three-, four-, five-, and six-membered rings respectively; similarly, GF-04 (e.g., carbonyl, amide, urea, sulfoxide, tert-butyl, and other) and GF-07 (sulfone, sulfonamide, phosphorus-containing moieties, etc.) describe the presence of many important functional groups. The same reasoning can be applied to the other subgraphs/generic fragments present in this illustration. The joint use of generic fragments and atom- or bond-based physicochemical properties (atomic weight, bond dipole moment, atomic molar refractivity, bond distance, atomic hydrophobicity, molecular accessibility, etc.) allow topological indices to discriminate the many diverse functional groups/moieties present in the molecules. Therefore, topological indices can be successfully applied to the physicochemical and structural characterization of large and heterogeneous datasets of chemicals. Topological indices (and consequently, the *MLIs* derived from them) can be used as inputs for the creation of highly predictive machine learning models (including the ones based on the PTML approach).

When applying PTML modeling for de novo drug design, the methodology now formally known as fragment-based topological design (FBTD) should be used [32,33,85]. In this sense, as indicated in its name, FBTD enables a deep physicochemical and structural interpretation of the topological indices used as inputs in linear and non-linear machine learning models (including those based on the PTML approach) [32,33,85]. The reason topological indices are essential for the FBTD methodology comes from the fact that they are fairly easy to calculate, they provide considerable information on different important 3D structural aspects (e.g., dihedral angles, molecular accessibility, volume, etc.) [86,87,88] despite having a 2D nature, and can be expressed as linear combinations of different generic fragments (GF) in a molecule [89,90,91,92,93,94,95] (Figure 3); the latter aspect enables the calculation of the quantitative contribution of any fragment to the biological effect under study.

Therefore, when using the FBTD methodology, the *MLIs* derived from topological indices in a PTML model are physicochemically and structurally interpreted; such interpretations lead to the chemistry-driven extraction and subsequent fusion/connection of suitable molecular fragments, ultimately yielding novel and rationally designed molecular entities with desired biological profiles (e.g., high activity, low toxicity, and/or enhanced pharmacokinetics) [29,30,31,32,33,34,96]. The upcoming ideas will focus on describing the cutting-edge works that have combined PTML modeling with FBTD for de novo drug design in MTDD-based anticancer research.

The first reports on the applications of PTML modeling for de novo design were a series of three works where the mt-QSAR-LDA models were created from fragment descriptors combined with *MLIs* derived from bond spectral moments. The first of these works aimed to accelerate the rational design of multi-cell inhibitors against four prostate cancer cell lines [97]. The mt-QSAR-LDA model was developed from a dataset containing 1668 cases, exhibiting *Sn* > 88% and *Sp* > 92%. By using the mt-QSAR-LDA model, the calculation of the quantitative contributions revealed suitable fragments with positive contributions to the inhibitory activity against the four prostate cancer cell lines. After applying FBTD, some of these molecular fragments were merged, yielding six novel (structurally related) molecules with multi-cell inhibitory potency against the prostate cancer cell lines under study. In the second work, the mt-QSAR-LDA model was intended to rationalize the design of versatile inhibitors against 13 different breast cancer cell lines with varying degrees of sensitivity to anti-breast cancer drugs [98]. This mt-QSAR-LDA model was constructed from a dataset formed by 2272 cases and achieved *Acc* > 90%. The use of the FBTD methodology and the posterior calculations of the quantitative activity contributions permitted the selection and subsequent connection/fusion of different molecular fragments; this led to the design of nine molecules, which were predicted by the mt-QSAR-LDA model as multi-cell inhibitors against the 13 breast cancer cell lines. The third work involved the generation of an mt-QSAR-LDA model as a tool to enable the design of anti-brain tumor agents against seven diverse brain tumor cell lines [99]. The mt-QSAR-LDA model built here from 1236 cases displayed *Acc* > 88%. The joint combination of FBTD and the fragment quantitative contribution provided the necessary information for the design of six novel (but structurally related) chemicals that were predicted to exhibit multi-cell inhibitory potency against the seven brain tumor cell lines.

A second series of works on PTML models for de novo drug design followed, and, in this case, both linear and non-linear PTML models were generated. This series consisted of two works where the following procedure was applied. Two mt-QSAR models were built; the linear (mt-QSAR-LDA) model employed fragment descriptors and *MLIs* calculated from bond spectral moments while the non-linear (mt-QSAR-ANN) model relied on *MLIs* derived from different global topological indices. The mt-QSAR-LDA model was used to (a) extract molecular fragments, (b) calculate the quantitative contribution of those fragments to the multi-cellular anticancer activity, and (c) design new molecules as potential multi-cell inhibitors against multiple cancer cell lines. The mt-QSAR-ANN model was employed to theoretically validate the molecules designed by the mt-QSAR-LDA model. Thus, in the first work of this second series, two mt-QSAR models were built from 1651 data points to predict and design multi-cell inhibitors of 10 colorectal cancer cell lines [100]. The mt-QSAR-LDA model exhibited *Acc* > 93%; for the mt-QSAR-ANN model, *Acc* > 92% was achieved. After applying the FBTD methodology and calculating the fragments’ quantitative contributions, nine molecules were designed by using the mt-QSAR-LDA model; these molecules were predicted as multi-cell inhibitors against the 10 colorectal cancer cell lines. The mt-QSAR-ANN model predicted the nine designed molecules as multi-cell inhibitors in at least 8 of the 10 colorectal cancer cell lines. The second work of this series enabled the design and prediction of chemicals with multi-cell anticancer activity against four bladder cancer cell lines [101]. The two mt-QSAR models reported in this work were developed from a dataset comprising 664 cases, exhibiting *Acc* > 92%. Here, the posterior physicochemical and structural interpretation of the descriptors (via the FBTD methodology) and subsequent computation of the quantitative contributions by the mt-QSAR-LDA model led to the merging of different suitable fragments. As a result, eight molecules were generated. The mt-QSAR-LDA model predicted the eight designed molecules to exhibit multi-cell inhibitory potency against the four bladder cancer cell lines. The same was confirmed by the mt-QSAR-ANN model, which predicted that 6 of the 8 designed molecules presented multi-cell inhibitory activity against the four bladder cancer cell lines while the other two design molecules were predicted as active against 3 of the four bladder cancer cell lines.

Two works were developed in the context of versatile inhibitors of proteins associated with breast cancer. In the first of them, research was conducted to create a PTML-LDA model for the fragment-based design and prediction of multi-target inhibitors of protein related to breast cancer [102]. In this sense, the PTML-LDA model was created from a dataset formed by 24,285 cases where chemicals were experimentally tested by considering at least 1 out of 2 *b_m_* (IC_50_ or the inhibition constant K_i_), at least 1 out of 19 breast cancer-related proteins (*t_s_*), and at least 1 out of 2 labels of assay information; in addition, the reliability of the assay was taken into account. The PTML-LDA model used *MLIs* derived from atom-bases quadratic indices and had very good performance, with *Sn* and *Sp* values higher than 93%. In this work, eight new molecules were designed by a combination of the PTML-LDA model and the FBTD methodology; the designed molecules were predicted as multi-target inhibitors against the 19 breast cancer-related proteins under study. The second work focused on building an mt-QSAR-ANN model to predict dual-target inhibitors of the proteins named cyclin-dependent kinase 4 (CDK4) and human epidermal growth factor receptor 2 (HER2) [103]. Here, the mt-QSAR-ANN model was built from a dataset containing 2213 data points and *MLIs*-based topological indices (path-based atomic connectivity indices and 2D-autocorrelations). The mt-QSAR-ANN model displayed with *Sn* > 86% and *Sp* > 75%. The application of FBTD led to the design of six molecules with half of them being predicted as dual target inhibitors of CDK4 and HER2.

**Figure 4:**
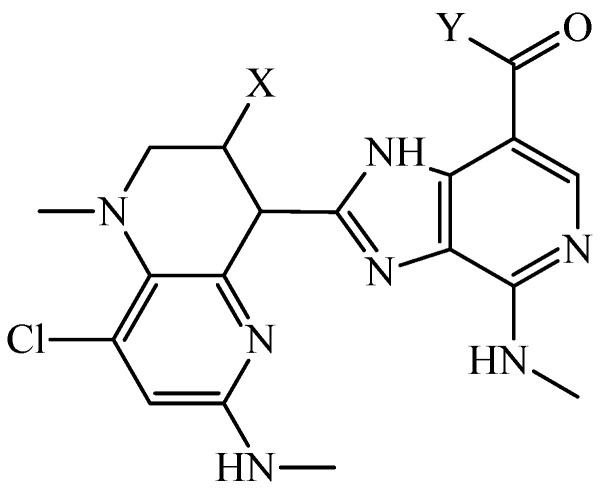
General structure of the two most promising multi-target inhibitors of BRD2, BRD3, and BRD4. The original codes of these molecules are ABD-001 and ABD-002. For the case of ABD-001, X = –NH_2_ and Y = (pyridin-2-yl)oxidanyl; for the case of ABD-002, X = (pyridin-2-yl)oxidanyl and Y = –NH_2_.

In a 2019 report, researchers focused their attention on the in silico design of multi-target inhibitors of the bromodomain-containing proteins 2, 3, and 4 (BRD2, BRD3, and BRD4, respectively) [104], which could lead to future versatile anticancer chemotherapies. In this report, two mt-QSAR models were developed from 1166 cases; the linear (mt-QSAR-LDA) used *MLIs* calculated from the total atom-based quadratic indices as the inputs while the non-linear model was an ensemble of neural networks (mt-QSAR-EL-ANN) using total atom-based quadratic indices as its inputs. The mt-QSAR-LDA model had *Sn* > 86% and *Sp* > 87%; for the mt-QSAR-EL-ANN model, *Sn* > 91% and *Sp* > 92% were obtained. Through the joint use of FBTD and the fragments’ quantitative contributions, it was possible to design six molecules. These molecules were predicted by both mt-QSAR models as triple target inhibitors of BRD2, BRD3, and BRD4. To provide another in silico perspective, molecular docking calculations were performed to explore the potential binding mechanisms of the designed molecules. Molecular docking calculations confirmed the triple-target profile of the designed molecules; the interaction determined by molecular docking matched the physicochemical and structural insights provided by the FBTD methodology. The two most promising molecules were predicted by docking to exhibit more favorable binding energies than the reference ligands present protein-ligand complexes determined by x-ray (Figure 4).

**Figure 5:**
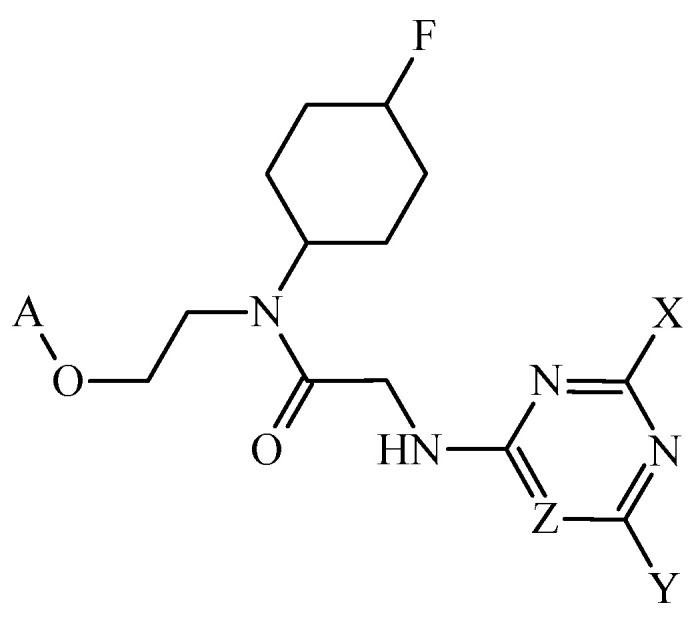
Chemical structure of three molecules designed as simultaneous multi-protein and multi-cell inhibitors against pancreatic cancer. The original codes of these molecules are MPMCI-001, MPMCI-002, and MPMCI-003. For the case of MPMCI-001, A = H, X = –NH_2_, Y = H, and Z = C. For MPMCI-002, A = H, X = Y = –NHCH_3_, and Z = C. For MPMCI-003, A = –CH_3_, X = –NHCH_3_, Y = H, and Z = N.

Three works were carried out on the applications of PTML modeling for de novo design in MTDD-based anticancer with emphasis on two of the cancers with the poorest prognoses: liver and pancreatic cancers. In this sense, these works mainly focused on designing chemicals with phenotypic multi-cell potency against different cancer cell lines. The first report aimed to discover multi-cell inhibitors of 17 liver cancer cell lines [105]. Here, an mt-QSAR-ANN model was built from 3079 data points and using *MLIs* derived from total and local atom-based quadratic indices as inputs. The mt-QSAR-ANN exhibited *Sn* > 80% and *Sp* > 85%. The FBTD methodology permitted the physicochemical and structural interpretation of the mt-QSAR-ANN and the subsequent direct extraction of the molecular fragments responsible for the increase of the multi-cell anticancer activity. As a result, eight molecules were designed, with six of them being predicted as multi-cell inhibitors against the 17 liver cancer cell lines. The second and third work were focused on PTML modeling applied to the design of anti-pancreatic cancer agents. In the second work, an mt-QSAR-EL-ANN model was developed to speed up the design of multi-cell inhibitors against 31 pancreatic cancer cell lines [106]. To do so, the mt-QSAR-EL-ANN model used 5797 data points and *MLIs* obtained from the stochastic and non-stochastic (total and local) atom-based quadratic indices. The mt-QSAR-EL-ANN model achieved *Sn* > 81% and *Sp* > 82%. Six novel molecules were designed through the application of the FBTD methodology; 4 out of six molecules were predicted by the mt-QSAR-EL-ANN model as multi-cell inhibitors against at least 28 out of 31 pancreatic cell lines. In the third work, two PTML models based on multilayer perceptron networks (PTML-MLP) were created by considering a dataset containing 9705 cases [107], which included two measures of activity endpoints *b_m_*, 34 targets *t_s_* (the 31 pancreatic cancer cell lines mentioned above and three pancreatic cancer-related proteins), and five labels of assay protocols *a_p_*. For the creation of the first of these PTML-MLP models, the *MLIs* derived from bond spectral moments and atom- and bond-based connectivity indices were used as inputs; the second PTML-MLP model was based on *MLIs* calculated from the atom-based local stochastic quadratic indices. Both PTML-MLP models displayed *Sn* > 78% and *Sp* > 84%. Although both PTML-MLP models were physicochemically and structurally interpreted, the FBTD methodology was applied only to the second of these PTML-MLP models. Consequently, the second PTML-MLP model was employed to design new molecules while the first PTML-MLP model was used as a filter to validate the design performed by the first model. Three molecules were designed and the three of them were predicted as multi-target inhibitors against pancreatic cancer (Figure 5); this means that both PTML-MLP models predicted the designed molecules as multi-protein inhibitors against the three pancreatic cancer-related proteins and multi-cell inhibitors against at least 30 of 31 pancreatic cancer cell lines.

**Figure 6:**
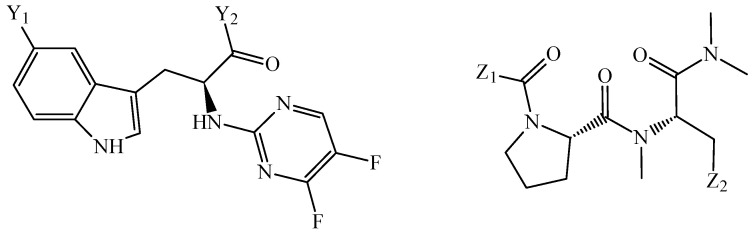
Two chemical families of molecules designed by combining a PTML-MLP model and FBTD. The original codes of these molecules were ASP-VALC-01, ASP-VALC-02, ASP-VALC-03, and ASP-VALC-04. The first chemical family (formed by ASP-VALC-01 and ASP-VALC-02) contained the substituents Y_1_ and Y_2_; Y_1_ = H and Y_2_ = 2-oxomorpholin-4-yl for the case of ASP-VALC-01 while Y_1_ = OH and Y_2_ = morpholin-4-yl for the case of ASP-VALC-02. The second chemical family (formed by ASP-VALC-03 and ASP-VALC-04) contained the substituents Z_1_ and Z_2_; Z_1_ = 2,6-difluorophenyl and Z_2_ = 4-hydroxyphenyl for the case of ASP-VALC-03 while Z_1_ = pyridin-3-yl and Z_2_ = 1H-indol-3-yl for the case of ASP-VALC-04.

Last, the joint use of PTML modeling and FBTD was reported for the de novo generation of multi-cell inhibitors against different lung cancer cell lines [108]. In this work, the PTML-MLP, which was created from fifteen *MLIs* and achieved *Sn* > 77% and *Sp* in the interval 79–87.8%, was able to classify/predict the anti-lung cancer activity of a dataset formed by 7379 cases of molecules against nine different lung cancer cell lines (of varying degrees of drug sensitivity to current anticancer drugs) while also considering information regarding the ability of each lung cancer cell line to be sensitivity to immunotherapy [109]. The interpretation of the PTML-MLP model, which was carried out by utilizing the FBTD methodology, permitted the analysis of diverse molecular fragments responsible for the multi-cell inhibitory activity against the nine lung cancer cell lines, leading to the design of four new molecules belonging to two different chemical families (Figure 6). The designed molecules we confirmed as multi-cell anti-lung cancer agents by both the developed PTML-MLP model and CLC-Pred 2.0 [110]; the latter is a state-of-the-art webserver, which was built to predict anticancer activity against a panel of the 60 most known cancer cell lines.

### 3.4. Future Perspectives on PTML Modeling

As demonstrated in the previous subsections, PTML modeling is very useful in both target-based and phenotypic drug discoveries. In fact, in the context of de novo drug design, a major hurdle is that the designed molecules have not been able to dock well in the protein pockets [111]; however, PTML modeling, when combined with the FBTD methodology, has evolved to the point where the designed molecules have been confirmed by well-established in silico approaches such as molecular docking [104,112], thus overcoming the aforementioned major hurdle.

In any case, there is always room for improvement and there are three main directions. One of them is, that, in the context of MTDD-based anticancer research, PTML modeling has focused mainly on the activity domain, providing deeper insights on the prediction and design of multi-protein and/or multi-cell inhibitors against many different types of cancer. Therefore, future efforts should be focused on developing mtk-QSBER models, which would enable the simultaneous prediction of multi-target anticancer activity while considering toxic effects and pharmacokinetic profiles at both in vitro and in vivo levels.

Second, PTML modeling combined with FBTD (see all the works reported in the previous subsection) has permitted to establish the theoretical foundations for the efficient computer-aided de novo design of chemicals with multi-protein and/or multi-cell inhibitory activity. To date, the majority of designed molecules have not yet reached experimental validation; thus, confirming the in vitro or in vivo anticancer efficacy of these novel molecules generated according to the de novo design paradigm remains a crucial next step for the field. Therefore, we recommend the synthesis and posterior biological evaluation to confirm the versatile anticancer activity of the designed molecules; this will consolidate PTML modeling (and subsequently, FBTD) as a powerful and innovative computational approach for antineoplastic discovery.

Last, the other main direction is that PTML modeling should also be devoted to exploring other chemical families beyond the realm of small organic molecules. This is the case of biosequence-based molecules such as peptides, micro-ribonucleic acids, and aptamers, which have emerged as potential therapeutic solutions in anticancer research [113,114,115].

## 4. Conclusions

Anticancer discovery continues to be an intensive research area, which requires the support of powerful in silico approaches to accelerate the discovery of versatile, effective, and safe MTDD-based chemotherapies. In this sense, how PTML models are mathematically conceived through the integration of chemical information with biological data, allows them to be used as tools for both virtual screening and de novo tasks across different biological endpoint measures, targets, and assay protocols. Consequently, we consider that PTML modeling considerably adds new and promising insights to the arsenal of in silico approaches for MTDD. We envisage that PTML modeling can be a great ally of modern drug development campaigns, rationalizing and prioritizing the identification/design of novel molecular entities in different MTDD scenarios.

## Abbreviations

The following abbreviations are used in this manuscript:

*Acc*	Accuracy
ANN	Artificial neural networks
*a_p_*	Assay conditions or protocols
*avg*[*X*]*e_j_*	Average value
*b_m_*	Biological effects
BRD2	Bromodomain-containing protein 2
BRD3	Bromodomain-containing protein 3
BRD4	Bromodomain-containing protein 4
CDK4	Cyclin-dependent kinase 4
*e_j_*	Experimental condition
FBTD	Fragment-based topological design
GF	Generic fragments
HER2	Human epidermal growth factor receptor 2
IC_50_	Half-maximal inhibitory concentration
LDA	Linear discriminant analysis
logP	Logarithm of the n-octanol/water partition coefficient
*MLIs*	Multi-label indices; these are also denoted as *D*[*X*]*e_j_* in Equation (2) of this article
mt-QSAR	Multi-target QSAR model
mt-QSAR-ANN	Multi-target QSAR model based on an artificial neural network
mt-QSAR-EL-ANN	Multi-target QSAR model based on an ensemble of artificial neural networks
mt-QSAR-LDA	Multi-target QSAR model based on linear discriminant analysis
mtc-QSAR	Multi-condition QSAR
mtc-QSAR-LDA	Multi-condition QSAR based on linear discriminant analysis
MTDD	Multi-target drug discovery
mtk-QSBER	Multi-tasking model for quantitative structure-biological effect relationships
mtk-QSBER-LDA	Multi-tasking QSBER model based on linear discriminant analysis
*n*(*e_j_*)	Number of chemicals that comply with a specific experimental aspect of *e_j_*
*Num*	Numerator, which can be the range (difference between the maximum and minimum values of *X*), the standard deviation of *X* values, or a value of 1
*p*(*e_j_*)	*A priori* probability of finding a chemical tested by considering a specific experimental aspect of *e_j_*
PSA	Polar surface area
PTML	Perturbation-theory machine learning
PTML-ANN	PTML model based on an artificial neural network
PTML-LDA	PTML model based on linear discriminant analysis
PTML-MLP	PTML model based on a multilayer perceptron network
QSAR	Quantitative structure-activity relationships
*t_s_*	Targets
RF	random forests
SMILES	Simplified molecular-input line-entry system
*Sn*	Sensitivity
*Sp*	Specificity
SPM	Statistical performance metrics
SVM	Support vector machines
*X*	Molecular descriptors
v_sm_	Numerical value for a particular SPM
*Y*	Exponent, which can take the values of −1, −0.5, 0, 0.5, or 1
